# Single cell oil production by *Trichosporon cutaneum* from steam-exploded corn stover and its upgradation for production of long-chain α,ω-dicarboxylic acids

**DOI:** 10.1186/s13068-017-0889-7

**Published:** 2017-08-23

**Authors:** Chen Zhao, Hao Fang, Shaolin Chen

**Affiliations:** 1 College of Life Sciences, Northwest A&F University, 22 Xinong Road, Yangling, 712100 Shaanxi China; 2 National Engineering Laboratory for Cereal Fermentation Technology, Jiangnan University, 1800 Lihu Avenue, Wuxi, 214122 Jiangsu China

**Keywords:** Single cell oil, *Trichosporon cutaneum*, Steam-exploded corn stover, On-site cellulase production, Three-stage enzymatic hydrolysis, α,ω-dicarboxylic acids, Self-metathesis

## Abstract

**Background:**

Single cell oil (SCO) production from lignocelluloses by oleaginous microorganisms is still high in production cost, making the subsequent production of biofuels inviable economically in such an era of low oil prices. Therefore, how to upgrade the final products of lignocellulose-based bioprocess to more valuable ones is becoming a more and more important issue.

**Results:**

Differently sourced cellulases were compared in the enzymatic hydrolysis of the steam-exploded corn stover (SECS) and the cellulase from the mixed culture of *Trichoderma reesei* and *Aspergillus niger* was found to have the highest enzymatic hydrolysis yield 86.67 ± 4.06%. Three-stage enzymatic hydrolysis could greatly improve the efficiency of the enzymatic hydrolysis of SECS, achieving a yield of 74.24 ± 2.69% within 30 h. Different bioprocesses from SECS to SCO were compared and the bioprocess C with the three-stage enzymatic hydrolysis was the most efficient, producing 57.15 g dry cell biomass containing 31.80 g SCO from 327.63 g SECS. An efficient and comprehensive process from corn stover to long-chain α,ω-dicarboxylic acids (DCAs) was established by employing self-metathesis, capable of producing 6.02 g long-chain DCAs from 409.54 g corn stover and 6.02 g alkenes as byproducts.

**Conclusions:**

On-site cellulase production by the mixed culture of *T. reesei* and *A. niger* is proven the most efficient in providing cellulase to the lignocellulose-based bioprocess. Three-stage enzymatic hydrolysis was found to have very good application value in SCO production by *Trichosporon cutaneum* from SECS. A whole process from corn stover to long-chain DCAs via a combination of biological and chemical approaches was successfully established and it is an enlightening example of the comprehensive utilization of agricultural wastes.

## Background

As an agricultural country with more than 1.3 billion people to feed, China has plenty of agricultural wastes or residues that should be utilized in an economic and environmentally friendly way. This is good for rural economy and environment protection because the reality is that those agricultural residues are always treated improperly, e.g., combusted directly, which causes serious environmental problem [1–3]. Bioconversion of them to fuels or chemicals, therefore, is promising and beneficial to the rural economy and environment. This kind of scientific research and relevant industry should be encouraged and supported as the growth rate of Chinese economy decreases but environmental burden increases.

Single cell oil (SCO) from microorganism is thought to be a desirable alternative oil source to plant oil or animal fat due to the high productivity, the low land requirement, as well as their particular and precise biochemical and physicochemical properties [4–6]. Many oleaginous microorganisms can accumulate high lipid content, some up to 80% dry cell weight or even higher [7]. Among them, *Trichosporon cutaneum* is a promising producer of SCO from lignocelluloses because of its high lipid yield and strong tolerance to inhibitors ubiquitously existing in the pretreated lignocellulosic materials [8, 9].

Pretreatment is still the most expensive single-unit operation in the lignocellulose-based bioprocesses [4, 10]. Steam explosion, which is the most commonly used pretreatment method and opined to be close to commercialization, can greatly improve the enzymatic digestibility of lignocellulosic materials [11–13]. Steam explosion produces lots of inhibitors that are resulted from the decomposition of hemicellulose and lignin [11, 14], thus making *T. cutaneum* a desirable candidate for SCO production from steam-exploded lignocellulosic materials.

The concept of on-site enzyme production has many advantages such as saving costs of separation, concentration, storage, and transportation [15, 16]. In addition, use of lignocellulosic biomass as substrate to induce cellulase production has an increased enzymatic hydrolysis specificity for the substrate itself than others [4, 17]. Mixed culture of *Trichoderma reesei* and *Aspergillus niger* is advantageous over the monoculture of *T. reesei* or *A. niger* in cellulase production and the enzymatic hydrolysis of steam-exploded corn stover (SECS) [12, 13].

Multi-stage enzymatic hydrolysis was found to be capable of improving the efficiency of enzymatic hydrolysis and the whole bioprocess, especially when high solid loading was used [4, 18, 19]. The volume of the multi-stage enzymatic hydrolysate is several times of one-stage enzymatic hydrolysate, leading to lower sugar concentration. The application value of multi-stage enzymatic hydrolysis, however, was proven in SCO production where high initial concentrations of fermentable sugars were unfavorable, unlike bioethanol production which prefers high sugar concentration [4, 18].

Single cell oil is a good starting material for biodiesel [6]. However, biodiesel is an imperfect final product nowadays because of the low oil prices, making it economically uncompetitive. Thus, transforming SCO to value-added chemicals is important to the commercialization of the lignocellulose-based bioprocess. Long-chain α,ω-dicarboxylic acids (DCAs), which are important platform chemicals and building blocks for biodegradable polymers [20], are much more valuable than biodiesel and other biofuels.

In this work, mixed culture of *T. reesei* and *A. niger* was established to produce and supply cellulase in the context of on-site enzyme production. Then SECS was enzymatically hydrolyzed by the cellulase from the mixed culture of *T. reesei* and *A. niger*. Differently sourced cellulases were compared in the enzymatic hydrolysis of SECS. Subsequently, the enzymatic hydrolysates of SECS were fermented by *T. cutaneum* for SCO production. The bioprocesses using different cellulases were compared to select the most efficient one. Moreover, the three-stage enzymatic hydrolysis was adopted to further enhance the efficiency of the bioprocess from SECS to SCO. Furthermore, the unsaturated fatty acids hydrolyzed from SCO were upgraded to long-chain DCAs via self-metathesis. We tried to seek the most efficient process of SCO production and the comprehensive utilization and conversion of corn stover to value-added chemicals.

## Methods

### Steam-exploded corn stover

The lignocellulosic material corn stover was from Kaifeng City, Henan Province, China. It was air-dried and stored at room temperature before use. Before steam explosion, it was sliced to a proper size (5–10 cm). The pretreatment of steam explosion was carried out in a 3.5 L reactor (Wuhai Gerun Environmental Protection Equipment Co., Ltd., China) under the following conditions: temperature 200 °C, pressure 1.6 MPa, pressure maintained duration 7 min, and substrate loading 100 g (dry material). The SECS was collected and washed 3 times using distilled water with a ratio of solid to liquid 1:10 (g:mL). Then the washed SECS residues were kept in refrigerator at 4 °C until further use. The composition of the washed SECS was as follows (dry material): glucan 52.5%, xylan 7.2%, lignin 22.8%, ash 11.4%, and others 6.1%.

### Microorganisms and media

*Trichoderma reesei* Rut-C30 and *A. niger* NL02 used for the mixed culture for cellulase production from SECS were obtained from the strain collection of the Department of Biochemical Engineering, Nanjing Forestry University. *T. cutaneum* ACCC20271, purchased from Agricultural Culture Collection of China, were used for SCO production from SECS enzymatic hydrolysate.

As for the seed medium for the preparation of *T. reesei* and *A. niger* inoculums and the fermentation medium for the cellulase production, please refer to our previous papers [4, 13]. These media were autoclaved at 121 °C for 20 or 30 min (20 min for the medium without SECS and 30 min for the medium with SECS).

Yeast peptone dextrose was used as seed medium for *T. cutaneum* and its composition was as follows (g/L): glucose 20, peptone 20, yeast extract 10. The fermentation medium for SCO production by *T. cutaneum* had the following composition: SECS enzymatic hydrolysate, (NH_4_)_2_SO_4_ in accordance with C/N ratio, KH_2_PO_4_ 3 g/L, MgSO_4_ 0.5 g/L, trace element solution 1%(v/v) and vitamin solution 0.1%(v/v). The composition of the trace element solution was as follows (g/L): EDTA 15, MnCl_2_·4H_2_O 1.0, CuSO_4_·5H_2_O 0.3, CaCl_2_·2H_2_O 4.5, NaMoO_4_·2H_2_O 0.4, H_3_BO_3_ 1.0, KI 0.1, CoCl_2_·6H_2_O 0.3, ZnSO_4_·7H_2_O 4.5 and FeSO_4_·7H_2_O 3.0. The components of the vitamin solution were 0.05 g/L d-biotin, 1 g/L calcium pantothenate, 1 g/L nicotinic acid, 1 g/L thiamine hydrochloride, 1 g/L pyridoxine hydrochloride, 0.2 g/L para-aminobenzonic acid, and 25 g/L (myo)inositol. The enzymatic hydrolysate was autoclaved at 121 °C for 30 min and the other solutions were sterilized by filtering through 0.22-μm membrane (Millipore, MA, USA). They were blended before use.

### Mixed culture of *T. reesei* and *A. niger*

For the mixed culture for cellulase production, 10% (v inoculum/v total volume) *T. reesei* and 10 or 2% (v inoculum/v total volume) *A. niger* inoculums were inoculated. The delay time of *A. niger* inoculation was 0 h (inoculated simultaneously), 24, or 48 h. These two conditions derived 6 mixed culture forms, denoted as 0 h/1:1, 0 h/5:1, 24 h/1:1, 24 h/5:1, 48 h/1:1, and 48 h/5:1. As to the details about the mixed culture, please refer to our previous work [12]. At least three parallel samples (*n* ≥ 3) were used in the analysis and data are shown in the form of means ± standard deviations.

## Enzymatic hydrolysis of SECS

### One-stage enzymatic hydrolysis of SECS

Both one- and three-stage enzymatic hydrolysis of SECS were conducted in 250-mL Erlenmeyer flasks with a working volume 50 mL containing 2.5 mL 1 M citrate buffer solution (pH 4.8), SECS, cellulase (added finally), and a supplementary amount of water to make up 50 mL. Once cellulase was added, flasks were incubated in an orbital shaker (140 rpm) at 50 °C for 48 or 72 h. Periodic sampling was done for analysis. At least three parallel samples (*n* ≥ 3) were used in the analysis and data are shown in the form of means ± standard deviations.

### Three-stage enzymatic hydrolysis of SECS

As for the three-stage enzymatic hydrolysis, the first, second, and third stages were conducted for 9, 9, and 12 h, respectively. Initial cellulase dosage was 15 FPIU/g glucan. At the end of each stage, the solid residue was separated by centrifugation (3000 rpm, 10 min). Fresh water and buffer were then added to the solid residue for the next stage enzymatic hydrolysis. To compensate the enzyme activity lost, 3 and 2 FPIU/g glucan fresh cellulase were added at the beginning of the second and the third stage, respectively. Thus the total cellulase dosage was 20 FPIU/g glucan. Periodic sampling was implemented for analysis. At least three parallel samples (*n* ≥ 3) were used in the analysis and data are shown in the form of means ± standard deviations.

The yield of enzymatic hydrolysis of SECS was calculated according to the following equation:

1 $${\text{Yield }}\left( \% \right) = ({\text{glucose}} + {\text{xylose}}) \, \left( {\text{g}} \right) \times 0. 9 \times 100/({\text{glucan}} + {\text{xylan}}){\text{ in substrate }}\left( {\text{g}} \right).$$

A conversion factor of 0.9 was used to eliminate the interfering effect because of the molecular weight changes of sugars before and after hydrolysis (a molecule of sugar without and with a molecule of H_2_O) so as to assure the accuracy. At least three parallel samples (*n* ≥ 3) were used in the analysis and data are shown in the form of means ± standard deviations.

### Fermentation of SECS enzymatic hydrolysate by *T. cutaneum*

The pre-culture was performed on the YPD medium at 28 °C and 150 rpm for 24 h. Then the pre-cultured *T. cutaneum* was inoculated into 250-mL Erlenmeyer flasks containing 50 mL the fermentation medium and incubated at 28 °C and 180 rpm with initial pH 6.0. Or the fermentation was carried out under the conditions we mentioned elsewhere. Sampling was conducted periodically for analysis to monitor the growth of *T. cutaneum* and the lipid accumulation during the fermentation process. At least three parallel samples (*n* ≥ 3) were used in the analysis and data are shown in the form of means ± standard deviations.

### SCO extraction from *T. cutaneum*

*Trichosporon cutaneum* cells were harvested by centrifugation and mixed thoroughly with 4 M HCl with a ratio of 6 mL 4 M HCl versus 1 g dry cell weight (DCW) by vortex. The mixtures were kept at room temperature for 30 min and then maintained in water bath at 100 °C for 3 min. Subsequently, they were cooled down quickly at −20 °C and added with a double volume of chloroform and methanol mixture (1:1 in volume ratio). Then, the mixtures were shaken completely at 5000 rpm for 5 min. The chloroform layer was collected, blended with an equal volume of 0.1% (w/v) NaCl solution, and vortexed for 5 min. The chloroform layer was collected and SCO was extracted by volatilizing chloroform. At least three parallel samples (*n* ≥ 3) were used in the analysis and data are shown in the form of means ± standard deviations.

### Self-metathesis reaction

The free fatty acids, hydrolyzed from SCO produced by *T. cutaneum* using acid-hydrolysis reaction and separated using physical method, were transferred into a three-necked round-bottomed flask. Then the flask was outgassed by purging with nitrogen gas for 0.5 h and added with the first-generation Grubbs catalyst or the second-generation Grubbs catalyst at a certain dosage so as to start the metathesis reaction. The catalysts were purchased from Sigma-Aldrich Co. LLC. The subsequent operations were performed according to the references [20, 21].

The unit of mol% was defined as 1 molar catalyst per 100 molar fatty acids. The conversion was calculated as follows:

2 $$\begin{aligned} {\text{Conversion }}\left( \% \right) &= \left( {\text{initial amount of fatty acids}}\right.\\ &\quad\left.- {\text{residual amount of fatty acids}} \right) \, \left( {\text{g}} \right) \\ & \quad \times 100/{\text{initial amount of fatty acids }}\left( {\text{g}} \right). \end{aligned}$$

The reaction was repeated at least three times. At least three parallel samples (*n* ≥ 3) were used in the analysis and data are shown in the form of means ± standard deviations.

## Analytical methods

### Determination of enzymatic activities of cellulase

Filter paper activity (FPA), beta-glucosidase activity (BGA), Avicelase activity, and CMCase activity were assayed in accordance with the standard method recommended by the International Union of Pure and Applied Chemistry (IUPAC) [22] with some modifications. The substrate used for assaying FPA was 50 mg (1 × 6 cm strip) Whatman No.1 filter paper (Kent, UK). The Unit (FPIU) of FPA was defined as the amount of enzyme needed for releasing 1 μmol of reducing sugars in 1 min. The substrate used for assaying BGA was ρNPG (*ρ*-nitrophenyl-β-d-1,4-glucopyranoside) (Sigma-Aldrich, St. Louis, MO, USA) and the Unit (IU) of FPA was defined as the amount of enzyme required to release 1 μmol of *ρ*-nitrophenol in 1 min. The substrates used for measuring Avicelase activity and CMCase activity were microcrystalline cellulose PH101 and carboxymethyl cellulose, respectively, purchased from Sigma-Aldrich, Co. LLC. The Unit (U) of Avicelase activity or CMCase activity was defined as the amount of enzyme required for generating 1 mg of reducing sugars in 1 h. For more details about the determination of enzymatic activities, consult our previous papers [1, 4]. At least three parallel samples (*n* ≥ 3) were used in the analysis and data are shown in the form of means ± standard deviations.

### Determination of monomeric sugars

Monomeric sugars were analyzed by Agilent 1100 (Agilent Technologies, Santa Clara, CA, USA) high-performance liquid chromatography (HPLC) (Biorad Aminex HPX-87P ion exclusion column). Deionized and degassed water was employed as the mobile phase at a flow rate of 0.6 mL/min. The column temperature was fixed at 55 °C. The eluate was detected by a refractive index detector. At least three parallel samples (*n* ≥ 3) were used in the analysis and data are shown in the form of means ± standard deviations.

### Determinations of dry cell biomass and SCO

Cell biomass was dried at 105 °C to a constant weight and measured by electronic balance. For SCO determination, cells (~30 mg biomass in 1 mL water solution), 4.5 mL of methanol (Sinopharm Chemical Reagent Co. Ltd., Shanghai, China), and 1 mL of tridecanoic acid (Sigma-Aldrich Co. LLC) as internal standard (approximately 0.5 mg/mL) were added into a tube. The tube was capped and vortexed for 30 s. Then a volume of 0.2 mL 12 M H_2_SO_4_ (Sinopharm Chemical Reagent Co. Ltd., Shanghai, China) was added and mixed by vortex. The tube was heated in water bath at 85 °C for 15 min for esterification. Then the tube was cooled down with tap water. Add 2 mL H_2_O and mix by vortex. Add 2 mL hexane (Sinopharm Chemical Reagent Co. Ltd., Shanghai, China) and mix again for fatty acid methyl esters (FAME) extraction. The hexane layer was collected and moved into vial for analysis.

The fatty acid composition was determined using capillary gas chromatography (GC). SP-2560 (100 m × 0.25 mm × 0.20 μm) capillary column (Supelco) was installed on a Hewlett Packard 5890 gas chromatograph equipped with a Hewlett Packard 3396 Series II integrator and 7673 controller, a flame ionization detector, and split injection (Agilent Technologies Inc., Santa Clara, CA, USA). The injector was kept at 260 °C, with an injection volume of 1 μL by split injection mode (ratio of 30:1). The initial oven temperature was set at 120 °C, then heated at a increasing rate of 3 °C/min to 240 °C and held for 20 min. The detector temperature was set at 250 °C. Helium was used as the carrier gas at a flow rate of 0.5 mL/min, and the column head pressure was 280 kPa. At least three parallel samples (*n* ≥ 3) were used in the analysis and data are shown in the form of means ± standard deviations.

### Determination of DCAs and other chemicals

The DCAs and byproducts resulted from the self-metathesis reaction were quantified with GC and characterized with GC–MS. DCAs and fatty acids were analyzed in the form of methyl ester using the method described by Miao et al. [23]. The equipment and conditions used for GC analysis were the same as described above. The GC–MS analysis employed Agilent 6890N Gas Chromatograph coupled with 5975B Mass Selective Detector and CDS Analytical Pyroprobe 500 Pyrolysis Injection Probe. The column was capillary column HP-5MS (30 m × 250 μm × 0.25 μm). The injector was kept at 260 °C, and 1 μL sample was loaded by split injection mode (ratio of 30:1). The initial oven temperature was set at 120 °C, then increased at a heating rate of 3 °C/min to 240 °C and held for 20 min. The detector temperature was 250 °C. Helium was used as the carrier gas at a flow rate of 0.5 mL/min. At least three parallel samples (*n* ≥ 3) were used in the analysis and data are shown in the form of means ± standard deviations.

## Results and discussion

### Mixed culture

**Fig. 1:**
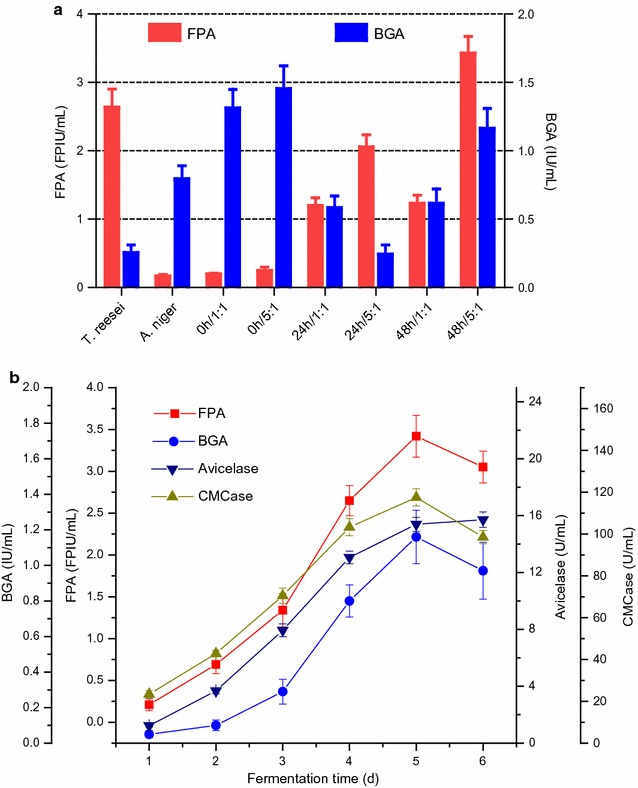
**a** FPAs and BGAs in the fermented broth obtained after 5 days fermentation from the monoculture of *T. reesei* or *A. niger* and the mixed cultures of *T. reesei* and *A. niger* grown in the medium containing steam-exploded corn stover (SECS). **b** Time course of cellulase production by the mixed culture (48 h/5:1) of *T. reesei* and *A. niger* induced by SECS. Data shown are means of at least three parallel samples (*n* ≥ 3) and *error bars* are standard deviations (mean ± SD)

Different mixed culture forms of *T. reesei* and *A. niger* were tried to identify the optimal form of cellulase production with the highest FPA and the bettered composition. Figure 1a shows the monoculture of *T. reesei* or *A. niger* and mixed cultures of *T. reesei* and *A. niger* after 5 days of cellulase production using SECS as the substrate and the inducer. It was found that the monoculture of *T. reesei* led to high FPA but extremely low BGA. This is because *T. reesei* is relatively complete in the composition of cellulase mixture but deficient in β-glucosidase [13, 24, 25]. Nonetheless, the monoculture of *A. niger* resulted in high BGA but very low FPA, mainly because *A. niger* is famous for its high BGA but not as robust as *T. reesei* in secreting a complete cellulase mixture that can degrade cellulose to monomeric sugars [12, 24].

The mixed culture forms 0 h/1:1 and 0 h/5:1 had the similar pattern to the monoculture of *A. niger*, except that the FPA values were slightly increased and BGA values were obviously enhanced. The BGAs of the mixture form 0 h/1:1 and 0 h/5:1 were higher than the monoculture of *A. niger*, indicating that the mix culture form 0 h/1:1 and 0 h/5:1 facilitated the β-glucosidase production. *A. niger* dominated in the mixed culture form 0 h/1:1 and 0 h/5:1. The mixed culture forms 24 h/1:1, 24 h/5:1, and 48 h/1:1 derived lower FPAs than the monoculture of *T. reesei* and BGAs than the monoculture of *A. niger*. This indicates that the competition between *T. reesei* and *A. niger* was too fierce to develop synergism. Consequently, these three mixed culture forms, 24 h/1:1, 24 h/5:1, and 48 h/1:1, are not suitable for cellulase production because the FPA was not adequately high.

The mixed culture form 48 h/5:1 had the highest FPA and relatively high BGA (Fig. 1a), indicating that the deficiency of *T. reesei* was overcome by mixed culture and the productivity was enhanced. The time course of the mixed culture form 48 h/5:1 is shown in Fig. 1b. All enzymatic activities increased as the fermentation process was underway and peaked after 5 days of fermentation, except Avicelase activity which continued increasing during the whole time course. The continuous increase in Avicelase activity may be because the main component of exoglucanases cellobiohydrolase I was driven by a strong promotor *Pcbh1* which manages cellobiohydrolase gene expression continuously [26]. Other enzymatic activities declined on Day 6 because *T. reesei* entered the phase of decline. Thus, the cellulase (FPA 3.42 ± 0.25 FPIU/mL, BGA 1.16 ± 0.15 IU/mL, Avicelase 15.39 ± 0.50 U/mL, and CMCase 117.51 ± 4.12 U/mL) was harvested after 5-d fermentation and applied to the subsequent experiment.

### Enzymatic hydrolysis of SECS

**Fig. 2:**
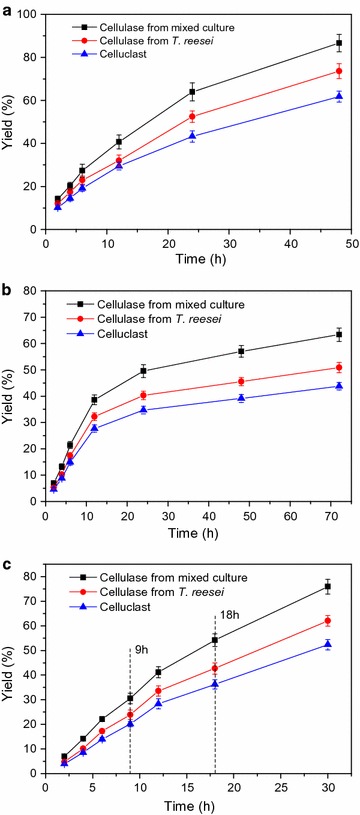
**a** One-stage enzymatic hydrolysis of SECS. The dosage of cellulase and the concentration of SECS (dry material) were 25 FPIU/g glucan and 100 g/L, respectively. **b** One-stage enzymatic hydrolysis of SECS. The dosage of cellulase and the concentration of SECS (dry material) were 30 FPIU/g glucan and 300 g/L, respectively. **c** Three-stage (9 + 9 + 12 h) enzymatic hydrolysis of SECS. The total dosage of cellulase and the concentration of SECS (dry material) were 20 FPIU/g glucan and 300 g/L, respectively. The initial cellulase loading was 15, and 3 and 2 FPIU/g of glucan fresh cellulase were added for the second and the third stage, respectively. All enzymatic hydrolysis experiments were conducted in 250-mL Erlenmeyer flasks with a volume of 50 mL. Data shown are means of at least three parallel samples (*n* ≥ 3) and *error bars* are standard deviations (mean ± SD)

Steam-exploded corn stover was hydrolyzed for the production of monomeric sugars using the cellulase from the mixed culture form 48 h/5:1. The commercial cellulase, Celluclast, and the cellulase from the monoculture of *T. reesei* were used as control. The results of the enzymatic hydrolysis of SECS are shown in Fig. 2a. After 48-h enzymatic hydrolysis, the yields of the cellulase from the mixed culture form 48 h/5:1, the cellulase from the monoculture of *T. reesei*, and the commercial cellulase were 86.67 ± 4.06%, 73.63 ± 3.46, and 61.73 ± 2.55 g/L, respectively. It was found that the cellulase from mixed culture 48 h/5:1 had better performance in the enzymatic hydrolysis of SECS than the cellulase from the monoculture of *T. reesei* when they were at the same dosage, 25 FPIU/g glucan. This is the same as the observation in our previous work [12, 13] and indicates that the composition of the cellulase was ameliorated by the mixed culture of *T. reesei* and *A. niger*. Moreover, both the cellulase from the mixed culture form 48 h/5:1 and the cellulase from the monoculture of *T. reesei* released higher concentration of glucose from SECS than the commercial cellulase, Celluclase, which was purchased from Sigma-Aldrich (St. Louis, MO, USA). This is because the use of lignocellulosic biomass as substrate to induce cellulase production has an increased enzymatic hydrolysis specificity for the substrate itself than others [15, 17, 27]. Therefore, the on-site cellulase production by mixed culture of *T. reesei* and *A. niger* is applicable and promising in the lignocellulose-based bioprocesses.

An enhancement in cellulase productivity and activities by the mixed culture of *T. reesei* and *A. niger* was reported by Ahamed and Vermette [28], but they did not test its performance in enzymatic hydrolysis. Some report found that the mixed culture of *T. reesei* and *A. phoenicis* had a lower FPA than the monoculture of *T. reesei*, but its cellulase had better performance than the cellulase from the monoculture of *T. reesei* and commercial cellulase in the enzymatic hydrolysis of lignocellulose [29]. Here, we obtained a mixed culture system with higher productivity of cellulase and better enzymatic hydrolysis performance.

In some lignocellulose-based bioprocesses such as bioethanol production, high solid loading is preferred because it could increase the product concentration and decrease the operating costs [18, 30]. The solid loading of 300 g SECS (dry material) was used as the substrate to produce higher concentration of glucose. The cellulase from the mixed culture form 48 h/5:1, the cellulase from the monoculture of *T. reesei* and the commercial cellulase were compared, and the results are shown in Fig. 2b. The cellulase from the mixed culture 48 h/5:1 still outperformed the other two cellulases when the solid loading was increased from 100 to 300 g/L (dry material). This also showed the superiority of the cellulase from the mixed culture over the cellulase from the monoculture of *T. reesei* and Celluclast. The yields after 48- and 72-h enzymatic hydrolysis by the cellulase from the mixed culture form 48 h/5:1 were only 57.03 ± 2.23 and 63.35 ± 2.53%, respectively, much lower than the previous enzymatic hydrolysis even when the cellulase dosage was 30 FPIU/g glucan which was higher than the cellulase dosage of 25 FPIU/g glucan used in the enzymatic hydrolysis of 100 g/L SECS.

Multi-stage enzymatic hydrolysis such as three-stage enzymatic hydrolysis is able to improve yield, shorten enzymatic hydrolysis time, and lessen cellulase dosage [18, 19]. Hence, it was used in this work for the enzymatic saccharification of 300 g SECS and the time course is shown in Fig. 2c. It was found that the efficiency of enzymatic hydrolysis was improved substantially. The time of the three-stage enzymatic hydrolysis was shortened from 72 to 30 h but the yield is higher than the one-stage enzymatic hydrolysis of SECS (Fig. 2b). The yield was able to reach 74.24 ± 2.69% by three-stage enzymatic hydrolysis just using 30 h, and a cellulase dosage of 20 FPIU/g. Three-stage enzymatic hydrolysis can to a great degree improve the efficiency of enzymatic hydrolysis, saving cellulase, and accelerating the enzymatic hydrolysis [18, 19].

In addition, the cellulase produced by the mixed culture of *T. reesei* and *A. niger* was superior to the cellulase produced by the monoculture of *T. reesei* and the commercial cellulase Celluclast in all the enzymatic hydrolysis processes in this work, suggesting that the composition of the cellulase was ameliorated, the synergism was enhanced and the degradation ability of the cellulase was strengthened. This work demonstrates that the mixed culture of *T. reesei* and *A. niger* we established is a good approach to realize on-site cellulase production and cellulase autarky. The enzymatic hydrolysates resulted from 100 and 300 g SECS were used as feedstock for the SCO production by *T. cutaneum*.

### Effects of culture conditions on SCO production by *T. cutaneum*

**Fig. 3:**
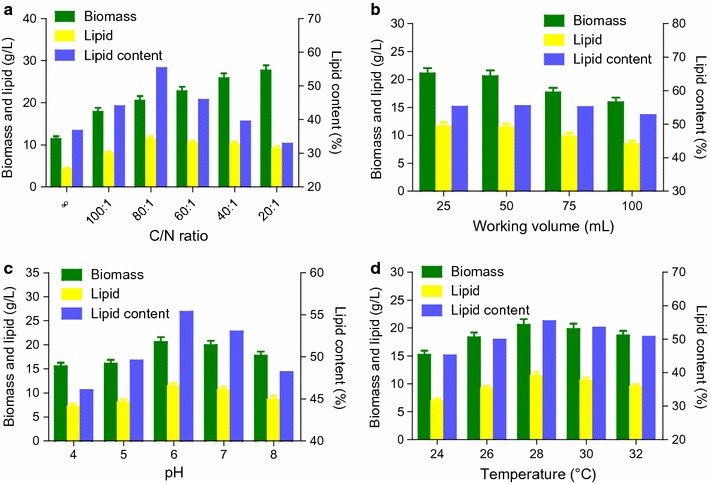
**a** Effect of C/N molar ratio on SCO production by *T. cutaneum*. “∞” means no nitrogen source, i.e., ammonium sulfate, was added. **b** Influence of working volume (mL) on SCO production by *T. cutaneum*. **c** Effect of pH on SCO production by *T. cutaneum* in the enzymatic hydrolysate of SECS. **d** Effect of temperature on SCO production by *T. cutaneum*. The enzymatic hydrolysate, used for SCO production, contained 50.84 ± 2.37 g/L glucose and 6.65 ± 0.32 g/L xylose. All the results were obtained after 8 days fermentation. Data shown are means of at least three parallel samples (*n* ≥ 3) and *error bars* are standard deviations (mean ± SD)

Different C/N ratios (carbon–nitrogen ratios) were compared to seek the most suitable one for SCO production by *T. cutaneum* in the enzymatic hydrolysate of SECS. The results of the fermentation for 8 days in the enzymatic hydrolysate containing 50.84 ± 2.37 g/L glucose and 6.65 ± 0.32 g/L xylose with the C/N ratios ranging from 20:1 to “∞” (without addition of nitrogen source) are presented in Fig. 3a. The molar C/N ratio 80:1 was found to be the best C/N ratio equal to the concentration of ammonium sulfate 1.58 g/L. If the C/N ratio was higher than 80:1, less cell biomass, lipid, and lipid content were produced. As the concentration of ammonium sulfate increased over 1.58 g/L, i.e., the C/N ratio decreased, the lipid content declined obviously because the lipid concentration decreased slightly but the cell biomass increased substantially. The results suggest that low C/N ratio is beneficial to cell growth but not to lipid accumulation, and that nitrogen source limitation rather than nitrogen source starvation facilitates SCO production. The best C/N ratio here is somewhat different from the work by Gao et al. [31] in which the C/N ratio about 50:1 was the best. This may be because we used different nitrogen sources and the type of nitrogen source affected SCO production [32]. In fact, the C/N ratio “∞” is not absolute because small quantity of organic nitrogen source exists in SECS enzymatic hydrolysate [31, 32], which was neglected in the calculation of the C/N ratio. That is the reason why *T. cutaneum* grew not so badly without addition of any nitrogen source. Therefore, the most suitable C/N ratio 80:1 was used into the subsequent experiments, where small quantity of organic nitrogen source in SECS was not taken into consideration and calculation.

Single cell oil production from monomeric sugars such as glucose and xylose is a process in great need of oxygen. Thus, the working volume affects the SCO production in shaking flasks and the results are shown in Fig. 3b. The more the working volume is, the lower the cell biomass, lipid, and lipid content produced. This makes sense because the oxygen transfer and supply cannot meet the increasing demand with the working volume increasing. However, the working volumes 25 and 50 mL led to almost the same results in terms of cell biomass, lipid, and lipid content. Hence, 50 mL was used as the working volume in the subsequent SCO production in shaking flasks.

Figure 3c shows the influence of initial pH on SCO production by *T. cutaneum* and it was found that the most proper pH was 6.0. The initial pH values higher or lower than that had negative effect on SCO production. The optimal pH value here is the same as the pH in the fermentation for SCO production by *T. cutaneum* reported by Qi et al. [9] but different from the work reported by Gao et al. [31] and Liu et al. [33] in which the pH value 5.0 was used. Nevertheless, the difference is not so large. Additionally, from Fig. 3c, we can know that the effect of pH on SCO production *T. cutaneum* is not very obvious. This indicates that *T. cutaneum* can tolerate wide range of pH. The pH resistance of *T. cutaneum* is probably innate owing to its exceptional habitats that range from industrial effluent to refinery waste. The initial pH value 6.0 was used in the subsequent experiments.

The effect of temperature on SCO production by *T. cutaneum* was studied and the results are shown in Fig. 3d. The optimal temperatures for *T. cutaneum* was 28 °C, which is the same as the report [9] but slightly lower than the reports [31, 33] in which 30 °C was used in the cultivation and fermentation. Actually, the difference between 28 and 30 °C was not that easy-to-see, though 28 °C was better which gave rise to the highest cell biomass, lipid, and lipid content. Accordingly, 28 °C was used in the following experiments.

### Fermentation of SECS enzymatic hydrolysates

**Fig. 4:**
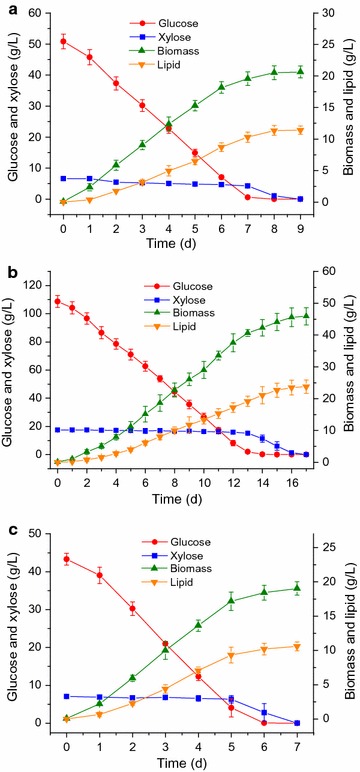
Time courses of SCO production by *T. cutaneum* in the enzymatic hydrolysates containing 50.84 ± 2.37 g/L glucose and 6.65 ± 0.32 g/L xylose (**a**), 108.65 ± 4.18 g/L glucose and 17.42 ± 0.85 g/L xylose (**b**), and 43.31 ± 1.57 g/L glucose and 7.06 ± 0.39 g/L xylose (**c**), respectively. Data shown are means of at least three parallel samples (*n* ≥ 3) and *error bars* are standard deviations (mean ± SD)

The enzymatic hydrolysates resulted from the different enzymatic hydrolysis processes (Fig. 2) were fermented by *T. cutaneum* to produce SCO. The results of SCO production are presented in Fig. 4. Figure 4a shows the time course of SCO production from the enzymatic hydrolysate resulted from the one-stage enzymatic hydrolysis of 100 g/L SECS, which contained 50.84 ± 2.37 g/L glucose and 6.65 ± 0.32 g/L xylose. *T. cutaneum* consumed almost all of glucose and xylose after 8 days of fermentation and produced 20.52 ± 1.09 g/L cell biomass and 11.35 ± 0.77 g/L lipid. The lipid content was 55.31%. The result is better than the work of Qi et al. [9], indicating that SECS enzymatic hydrolysate is the suitable substrate of *T. cutaneum* for SCO production which was also proven to be the suitable substrate of *Mortierella isabellina* for SCO production in our previous research [4]. Same as *M. isabellina*, *T. cutaneum* consumed glucose first and then xylose. This phenomenon is the same as the report by Gao et al. [31] but different from the report by Qi et al. [9] in which *T. cutaneum* seems to ferment glucose and xylose simultaneously.

When the solid loading of SECS increased from 100 to 300 g/L, the concentrations of glucose and xylose reached 108.65 ± 4.18 and 17.42 ± 0.85 g/L, respectively, after 72-h enzymatic hydrolysis, although the enzymatic hydrolysis yield was low. The high concentrations of fermentable sugars are beneficial to industrial applications such as bioethanol production because this could increase the concentrations of products and reduce the production cost [34]. Figure 4b shows the time course of SCO production by *T. cutaneum* in the SECS enzymatic hydrolysate containing 108.65 ± 4.18 g/L glucose and 17.42 ± 0.85 g/L xylose. It took more than 16 days for *T. cutaneum* to consume up all glucose and xylose. *T. cutaneum* produced 45.58 ± 2.95 g/L dry cell biomass and 23.49 ± 2.33 g/L lipid after 16 days of fermentation. The fermentation rate and lipid productivity are as good as the fermentation of the SECS enzymatic hydrolysate containing 50.84 ± 2.37 g/L glucose and 6.65 ± 0.32 g/L xylose. However, too long fermentation period is not viable in industry because it has bigger risks of contaminations and needs much stricter fermentation process control, which means higher production cost.

Although three-stage enzymatic hydrolysis can improve the enzymatic hydrolysis efficiency, it has apparent shortcomings, one of which is lower concentrations of fermentable sugars. Surprisingly, this instead is an advantage in the SCO production by *M. isabellina* where high initial concentrations of sugars are unfavorable [4]. Here, we applied the three-stage enzymatic hydrolysis in the SCO production by *T. cutaneum* to investigate its influence on the whole bioprocess from SECS to SCO. The enzymatic hydrolysate resulted from the three-stage enzymatic hydrolysis of SECS, which contained 43.31 ± 1.57 g/L glucose and 7.06 ± 0.39 g/L xylose, were fermented by *T. cutaneum* for SCO production and the time course is shown in Fig. 4c. *T. cutaneum* exhausted all glucose and xylose within 7 days. After 7 days fermentation, 19.05 ± 0.98 g/L dry cell biomass and 10.60 ± 0.65 g/L lipid were produced. It had the shortest fermentation period and the highest lipid content, 55.64%. This indicates that *T. cutaneum*, like many other oleaginous microbes, prefers low substrate concentration when carrying out fermentation for SCO production. In addition, it was found in Table 1 (the data of biomass productivity) that the growth rate of *T. cutaneum* in lower sugar concentration is similar to that in higher sugar concentrations. Therefore, lower sugar concentration is better than higher sugar concentration for SCO production.

**Table 1:** Results of single cell oil (SCO) production by *T. cutaneum* from steam-exploded corn stover (SECS)

Bioprocess	*A*	*B*	*C*
SECS (g dry material)	100 + 11.51	300 + 41.45	300 + 27.63
Glucose (g/L)	50.84 ± 2.37	108.65 ± 4.18	43.31 ± 1.57
Xylose (g/L)	6.65 ± 0.32	17.42 ± 0.85	7.06 ± 0.39
Volume of enzymatic hydrolyzate (L)	1	1	3
Fermentation time (d)	8	16	7
Biomass (g/L dry cell biomass)	20.52 ± 1.09	45.58 ± 2.95	19.05 ± 0.98
Biomass yield (g/g glucose + xylose)	0.357	0.362	0.378
Biomass productivity (g/L/d)	2.565	2.849	2.721
Total yield of biomass (g/g SECS)	0.184	0.133	0.174
Lipid (g/L)	11.35 ± 0.77	23.49 ± 2.33	10.60 ± 0.65
Lipid yield (g/g glucose + xylose)	0.197	0.186	0.210
Lipid productivity (g/L/d)	1.419	1.468	1.514
Lipid content (%)	55.31	51.54	55.64
Total yield of lipid (g/g SECS)	0.102	0.069	0.097
Total time from SECS to SCO (h)	240	456	198
Total productivity of biomass (g/h)	0.086	0.100	0.289
Total productivity of lipid (g/h)	0.047	0.052	0.161
Enzyme input (FPIU/g lipid)	115.64	201.15	99.06
Handling capacity (g SECS/h)	0.465	0.749	1.655
Utilization ratio of SECS (%)	86.67 ± 4.06%	63.35 ± 2.53%	75.92 ± 2.96%

*Trichosporon cutaneum* spent about 8, 16, and 7 days in fermenting the enzymatic hydrolysate resulted from the one-stage enzymatic hydrolysis of 100 g SECS, the enzymatic hydrolysate resulted from the one-stage enzymatic hydrolysis of 300 g SECS, and the enzymatic hydrolysate resulted from the three-stage enzymatic hydrolysis of 300 g SECS, respectively. From the standpoint of application, the enzymatic hydrolysate resulted from the three-stage enzymatic hydrolysis of SECS containing lower concentrations of fermentable sugars was the most suitable substrate for *T. cutaneum* in the context of SCO production. Multi-stage enzymatic hydrolysis such as the three-stage one is able to enhance the efficiency of enzymatic saccharification, shortening the enzymatic hydrolysis period, lessening the enzyme dosage, and improving the enzymatic hydrolysis yield [4, 18, 19]. However, it has a very obvious disadvantage, it produces several-fold more hydrolysate and lowers the product concentration [18, 19], rendering it not a good option in many bioprocesses. Here, the disadvantage becomes the advantage. Therefore, the three-stage enzymatic hydrolysis was found to be applicable in the SCO production by *T. cutaneum*.

### Comparison of different bioprocesses from SECS to SCO

**Fig. 5:**
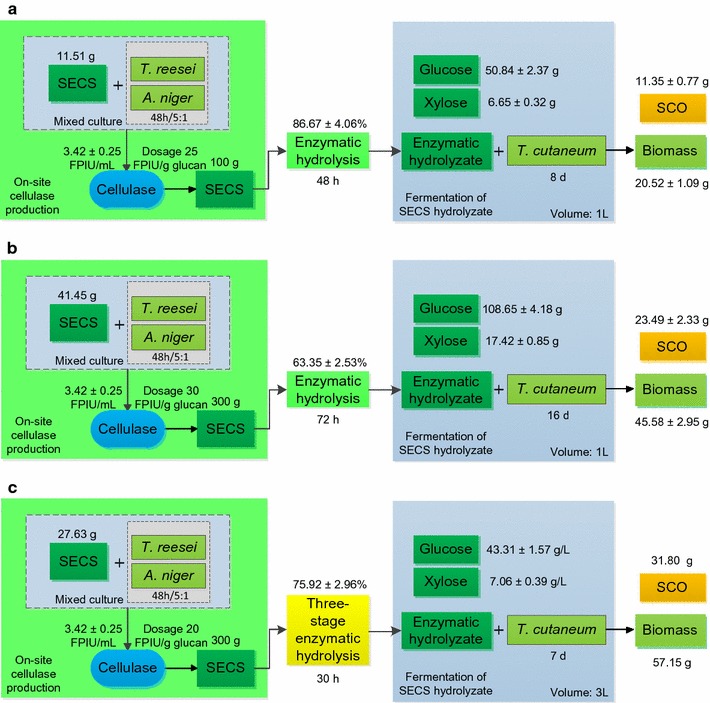
Comparison of the different bioprocesses from SECS to SCO in the context of on-site cellulase production, **a** A from 100 g dry SECS to SCO with one-stage enzymatic hydrolysis; **b** B from 300 g dry SECS to SCO with one-stage enzymatic hydrolysis; and **c** C from 300 g dry SECS to SCO with three-stage enzymatic hydrolysis

Different bioprocesses from SECS to SCO were compared comprehensively and they were outlined in Fig. 5. The detailed information about these bioprocesses is listed in Table 1. The SECS used for the whole process is the sum of the SECS used as substrate for the enzymatic hydrolysis to produce fermentable sugars and the SECS used for on-site cellulase production. The total amounts of the SECS for the bioprocess *A*, *B*, and *C* were 111.51, 341.45, and 327.63 g (dry material), respectively. The bioprocess *A* produced 1 L SECS enzymatic hydrolysate containing 50.84 ± 2.37 g/L glucose and 6.65 ± 0.32 g/L xylose from 100 g SECS by one-stage enzymatic hydrolysis. The bioprocess *B* produced 1 L SECS enzymatic hydrolysate containing 108.65 ± 4.18 g/L glucose and 17.42 ± 0.85 g/L xylose from 300 g SECS by one-stage enzymatic hydrolysis. The bioprocess C produced 3 L SECS enzymatic hydrolysate containing 43.31 ± 1.57 g/L glucose and 7.06 ± 0.39 g/L xylose from 300 g dry SECS by three-stage enzymatic hydrolysis.

These enzymatic hydrolysates of SECS were then fermented by *T. cutaneum* to produce SCO. The fermentation time for the bioprocess A, B, and C were 8, 16, and 7 days, respectively. The bioprocess C had the shortest fermentation period because it had the lowest starting sugar concentration. For the cell biomass and lipid, the bioprocess *C* is similar to the bioprocess *A*. The former one’s cell biomass and lipid were 19.05 ± 0.98 and 10.60 ± 0.65 g/L, respectively, and the latter one’s cell biomass and lipid were 20.52 ± 1.09 and 11.35 ± 0.77 g/L, respectively. In addition, the yields and productivities of the bioprocess *C* and *A* were close to each other (Table 1). They also had no big difference from those of the bioprocess *B*. This suggests that the different starting sugar concentrations just changed the fermentation time but made no big difference in other fermentation parameters.

Taking enzymatic hydrolysis and fermentation together into consideration, however, the bioprocess *B* had the lowest efficiency but the bioprocess *C* had the highest efficiency. The total time from SECS to SCO included the time for enzymatic hydrolysis and the time for SCO production, irrespective of the time for enzyme production or others. The bioprocess *C* had the shortest one 198 h while the bioprocess *B* had the longest one 456 h. Additionally, the bioprocess *C* had the highest total productivities of cell biomass and lipid, which were 0.289 and 0.161 g/h, respectively. Meanwhile, the bioprocess C had the lowest enzyme input 99.06 FPIU/g lipid and the highest handling capacity 1.655 g SECS/h. These results were achieved by the bioprocess C under the premise that the enzymatic hydrolysis yield was not so high, which was only 75.92 ± 2.96%. The results indicate that the efficiency of the bioprocess from SECS to SCO was greatly improved by the three-stage enzymatic hydrolysis. This work proved the application value of three-stage enzymatic hydrolysis, which had not been pointed out by the previous work on the establishment of multi-stage enzymatic hydrolysis [18, 19]. The bioprocess *C* is opined to be the most efficient one (Fig. 5c).

We succeeded in applying the three-stage enzymatic hydrolysis to the SCO production process from lignocellulose by *T. cutaneum* to enhance the efficiency, as we did before in the SCO production by *M. isabellina* [4]. As a whole, *T. cutaneum* outperformed *M. isabellina* in the SCO production from SECS. The former had higher fermentation rate, cell biomass, and lipid productivities, though the lipid content was slightly lower than the latter under same circumstances.

### Self-metathesis for production of long-chain DCAs

The SCO produced from SECS by *T. cutaneum* was hydrolyzed to produce fatty acids and among them the unsaturated fatty acids were transformed by self-metathesis to long-chain DCAs. The self-metathesis of the unsaturated fatty acids from the SCO was carried out under a nitrogen atmosphere by using first- or second-generation Grubbs catalyst. The proportions of unsaturated fatty acids of the SCOs from the different bioprocesses are presented in Table 2. There is no difference in the composition of the fatty acids among the SCOs produced via the different bioprocesses established and compared previously. The unsaturated fatty acids from the SCOs produced via the bioprocess C were used as the substrate of self-metathesis for the production of DCAs.

**Table 2:** Fatty acid compositions (%) of single cell oil produced by *Trichosporon cutaneum* via the bioprocess *A*, *B*, and *C*

Fatty acid	Structure	*A*	*B*	*C*
Palmitic	C16:0	27.68 ± 1.53	27.55 ± 1.38	25.98 ± 1.05
Palmitoleic	C16:1Δ9	1.38 ± 0.09	1.27 ± 0.15	1.57 ± 0.12
Stearic	C18:0	10.07 ± 0.41	10.66 ± 0.54	10.18 ± 0.49
Oleic	C18:1Δ9	50.25 ± 2.14	49.22 ± 1.05	51.25 ± 1.82
Linoleic	C18:2Δ9,12	8.32 ± 0.38	8.09 ± 0.87	8.82 ± 0.43
γ-Linolenic	C18:3Δ6,9,12	0.63 ± 0.08	0.57 ± 0.06	0.69 ± 0.05
Others		1.67	2.64	1.51

The unit of the compositions of fatty acids is %

Data shown are means of at least three parallel samples (*n* ≥ 3) and error bars are standard deviations (mean ± SD)

The effect of catalyst on self-metathesis was investigated and the result is shown in Table 3. It was found that the second-generation Grubbs catalyst was more efficient than the first-generation Grubbs catalyst in catalyzing the self-metathesis. The catalyst dosage of 0.1 mol% was the most appropriate because the resulted conversion 81.15 ± 2.71% was acceptable. Ten times more catalyst 1 mol% just improved the conversion by less than 10% and Grubbs catalysts are still expensive in market. Further work should be done to improve the efficiency of metathesis catalyst and reduce the cost. Some new Schrock-type and Grubbs-type catalysts have been invented [35, 36] but they are very expensive. Thus designing more efficient and cheaper catalyst is greatly needed because this decides the future of metathesis in DCAs production and other industrial applications.

**Table 3:** Effect of different catalysts dosage on the self-metathesis reaction

Catalyst	Dosage (mol %)^a^	Reaction time (h)	Conversion^b^ (%)
Grubbs 2nd	0.01	24	28.77 ± 0.76
Grubbs 2nd	0.1	1.5	81.15 ± 2.71
Grubbs 2nd	1	0.5	90.08 ± 3.25
Grubbs 1st	0.1	6	14.01 ± 0.49

Data shown are means of at least three parallel samples (*n* ≥ 3) and error bars are standard deviations (mean ± SD)

^a^ mol% is the unit defined as the molar quantity of catalyst per 100 g substrate

^b^Conversion (%) = (initial amount of fatty acids − residual amount of fatty acids) (g) × 100/initial amount of fatty acids (g)

**Fig. 6:**
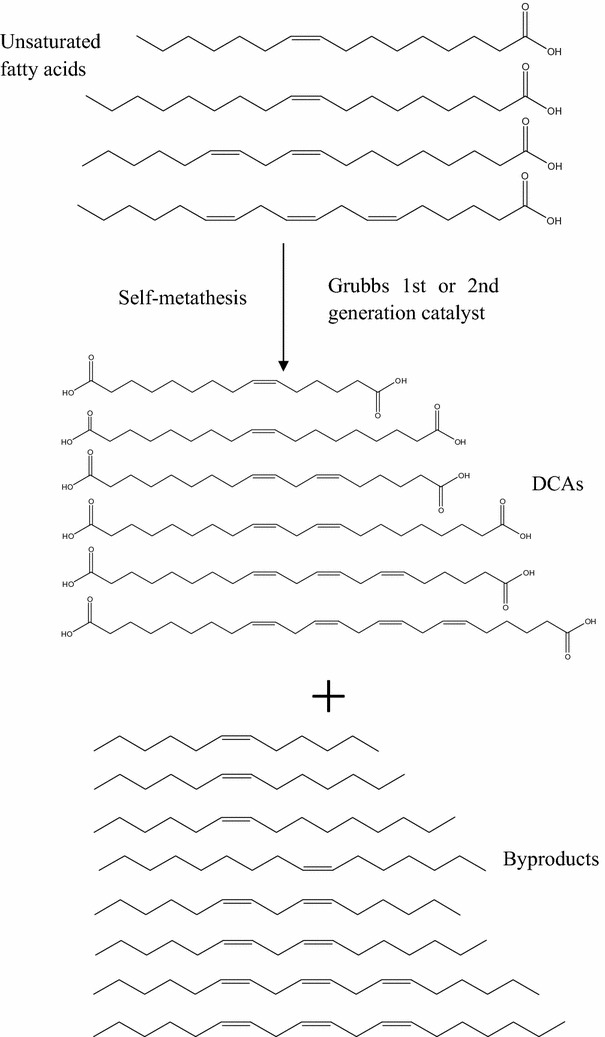
The detail of the self-metathesis reaction

The mechanism of the self-metathesis is illustrated in Fig. 6, which was deduced according to the theory of olefin metathesis. The double bonds “=” in the unsaturated fatty acids from the microbial lipids participated in the self-metathesis reaction, leading to new molecules with two carboxyl groups at both ends, i.e., DCAs [35, 36]. Moreover, the reaction produced alkene molecules which could be good feedstock for biofuels after some modifications to remove double bonds “=” [37, 38]. It is roughly estimated that half of the total amount of the unsaturated acids converted were transformed into long-chain DCAs and the other half were converted to byproducts. The products DCAs and the byproducts alkenes were identified by gas chromatography. So the mechanism deduced was proven by this work. Ngo et al. employed self-metathesis to produce DCAs from plant oils [20, 21]. However, plant oils are not so renewable as SCO. Therefore, this work made substantial advancement in producing DCAs, establishing a bioprocess from corn stover to DCAs and improving the efficiency.

**Fig. 7:**
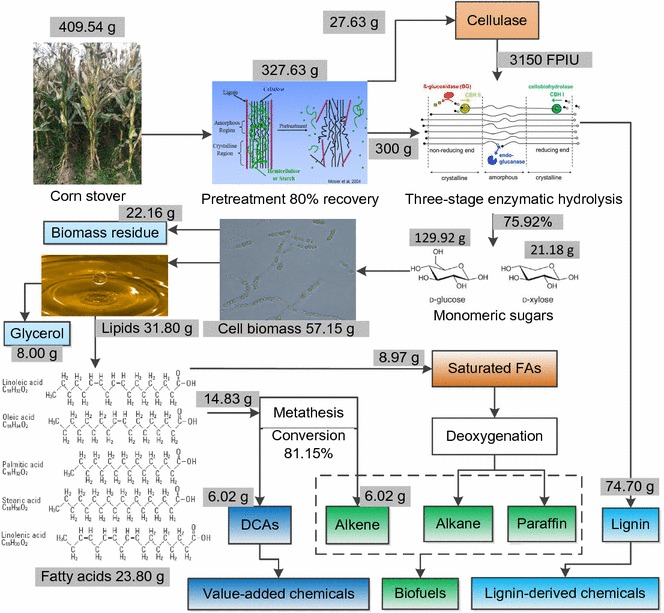
Overview of the processes from corn stover to long-chain DCAs, biofuels, and other products

The overall process from corn stover to DCAs is outlined and illustrated in Fig. 7, where the mass balance of the whole process was estimated. As shown in Fig. 6, 409.54 g corn stover (dry material) formed 327.63 g SECS (dry material) by the pretreatment of steam explosion in which the recovery was assumed to be 80%. The recovery assumption was based on our previous work on SECS, which always fluctuated between 70 and 90% (data not shown). Of 327.63 g SECS, 27.63 g SECS was used as the inducer of cellulase production by the mixed culture of *T. reesei* and *A. niger* and 300 g SECS was used as the substrate of enzymatic hydrolysis for production of fermentable sugars. The three-stage enzymatic hydrolysis produced 129.92 g glucose and 21.18 g xylose from 300 g SECS, the main fermentable sugars in the 3 L SECS enzymatic hydrolysate. Then the fermentable sugars were fermented and converted by *T. cutaneum* to yield 57.15 g dry cell biomass containing 31.80 g lipids. Subsequently, the lipids were extracted from *T. cutaneum* cells and hydrolyzed to form 23.80 g fatty acids, of which 14.83 g unsaturated fatty acids participated the self-metathesis reaction. After self-metathesis, 6.02 g long-chain DCAs and 6.02 g alkenes (Figs. 6, 7) were produced. In summary, 409.54 g corn stover could produce 6.02 g long-chain DCAs, 6.02 g alkenes, 8.97 g saturated fatty acids, 8.00 g glycerol, and 74.70 g lignin.

Figure 7 presents the outline of the process from corn stover to long-chain DCAs and the envisioned processes to make good use of the products and byproducts to produce value-added chemicals and fuels, strengthening the commercialization potential of the lignocellulose-based process. Long-chain DCAs are highly valuable platform chemicals as we aforementioned, which could be used as building blocks for a wide range of chemicals in industry [39, 40]. The saturated fatty acids, together with the byproducts of self-metathesis, could be transformed to biofuels [37, 38]. In addition, the side product lignin could also be transformed to valuable lignin-derived chemicals such as guaiacol and catechol [41]. This work provides a comprehensive way to utilize lignocellulose.

Here, we first report the whole process from corn stover to DCAs via a combination of the biological and chemical processes. Although some researchers reported the bioprocess from monomeric sugars to DCAs using engineered yeast [39], its controllability, efficiency, and versatility are not comparable with metathesis. Furthermore, no further advancement in that research has been reported. In short term, therefore, chemical routes are still dominant and irreplaceable because of their own merits. In long term, however, we look forward to combining the whole process from lignocellulose to DCAs or other chemicals into one step using lignocellulolytic microorganisms such as *T. reesei* and *Neurospora crassa* in the light of consolidated bioprocessing with the techniques of metabolic engineering and genome editing developing.

## Conclusions

The mixed culture of *T. reesei* and *A. niger* was a good approach to enhance cellulase production and improve enzymatic hydrolysis of pretreated corn stover. In addition, the cellulase produced by the mixed culture outperformed other cellulases. The application value of the three-stage enzymatic hydrolysis was confirmed in the SCO production from SECS by *T. cutaneum*, which could improve the efficiency of the bioprocess. Then the unsaturated fatty acids from SCO were upgraded to long-chain DCAs by self-metathesis. A whole process from corn stover to long-chain DCAs via a combination of biological and chemical approaches was successfully established to comprehensively utilized lignocellulose to produce value-added chemicals. This work is an enlightening example of the comprehensive utilization of agricultural wastes.

## Abbreviations

BGA: beta-glucosidase activity
CMC: carboxymethyl cellulose
C/N: ratio of carbon to nitrogen
DCA: α,ω-dicarboxylic acids
DCW: dry cell weight
FAME: fatty acid methyl esters
FPA: filter paper activity
GC: gas chromatography
GC–MS: gas chromatography–mass spectroscopy
HPLC: high-performance liquid chromatography
IU: international unit
IUPAC: International Union of Pure and Applied Chemistry
SCO: single cell oil
SECS: steam-exploded corn stover
YPD: yeast peptone dextrose
